# High crop load and low temperature delay the onset of bud initiation in apple

**DOI:** 10.1038/s41598-019-54381-x

**Published:** 2019-11-29

**Authors:** Julian Kofler, Anton Milyaev, Filippo Capezzone, Slobodan Stojnić, Nikola Mićić, Henryk Flachowsky, Magda-Viola Hanke, Jens-Norbert Wünsche

**Affiliations:** 1 Institute of Crop Science, Dept. of Crop Physiology of Specialty Crops (340f), University of Hohenheim, Stuttgart, Germany; 2 Institute of Crop Science, Dept. of Biostatistics (340c), University of Hohenheim, Stuttgart, Germany; 3 Faculty of Agriculture, University of Banja Luka, Banja Luka, Bosnia and Herzegovina; 4 Institute for Breeding Research on Fruit Crops, Julius Kühn-Institut (JKI), Federal Research Centre for Cultivated Plants, Dresden, Germany

**Keywords:** Statistical methods, Shoot apical meristem, Flowering

## Abstract

The reproductive cycle of apple (*Malus* × *domestica* Borkh.) starts with the induction of floral development, however, first morphological changes within the bud appear during the following period of bud initiation. This study identifies the onset and duration of bud initiation in the apple cultivars ‘Fuji’ and ‘Gala’, characterized by biennial and non-biennial bearing behaviour, respectively, and describes the effect of crop load and heat accumulation on the temporal pattern of floral development. The onset of flower bud initiation in heavy cropping ‘Gala’ trees was delayed for 20 days compared to trees with no crop load, but the rate of initiation was not affected by crop load. Bud initiation on heavy cropping ‘Fuji’ trees was minor, whereas trees with no crop load started initiating buds 19 days earlier than those of ‘Gala’ despite the same cropping status and growing degree hours in a given year. The onset of bud initiation in ‘Fuji’ ‘off’ trees was 5 and 20 days after summer solstice, respectively, in two consecutive growing seasons, suggesting that this process is driven by heat accumulation rather than by daylength. The results indicate, that the genetic make-up of the cultivar determines the onset of bud initiation. This can be delayed by increasing crop loads and low temperatures at the beginning of the flower formation process.

## Introduction

The two key factors determining crop load in apple (*Malus* × *domestica* Borkh.) are flower density and fruit set^1^. Since fruit set can be adjusted to some extent by flower or fruitlet thinning, the critical factor is the number of flower buds per tree^2^. Fruit growers aim for stable numbers of flower buds across years to reduce the risk of triggering the phenomenon of biennial bearing^3^. This cropping irregularity is characterized by large yields of small-sized fruit in ‘on’ years and low yields of over-sized fruit in ‘off’ years^4^, i.e. trees with ‘on’-bearing status change to ‘off’-bearing status in the subsequent year and vice versa. Apple cultivars differ in their degree of biennial bearing behaviour, e.g. ‘Gala’ has a regular bearing habit, whereas ‘Fuji’ shows a strong tendency to bear biennially^5^. Besides apple, biennial or alternate bearing is also commonly found in other fruit trees such as pistachio, pecan, olive, citrus, avocado or mango^6^. The physiological reason for entering an ‘off’ year is supposedly the competitive overlap of flower bud formation for the subsequent season and fruit development during the current season^7,8^.

Perennial fruit trees such as apple start their reproductive development early in the first growing season of a 2-year-cycle with inducing flower buds at the first stage of the flower bud formation process^9^. During this stage, termed flower induction, the vegetative meristem perceives a specific signal that either promotes flower bud development by triggering various biochemical processes or suppresses factors that cause the meristem to remain in a vegetative state^10^. For example, as recently shown^11^, sucrose could act as a signal molecule for mediating flower induction in apple. Several studies reported that high crop load inhibits flower induction in apple, leading to poor return bloom in the following year^6,12–15^. The exact mechanism of crop load-induced inhibition of flower induction still remains unclear, although there is good evidence that a high yield reduces next year’s flower density, hence crop load, in a specific way: mobile signals formed by developing fruit or specifically seed within the fruit (e.g. plant hormones such as gibberellins)^16–18^ or lack of certain nutrients (e.g. carbohydrates)^6^ inhibit the nearby bud meristem. Moreover, high fruit load and high levels of gibberellins are accompanied by an increased expression of the *TFL* gene^19,20^ and reduced expression of *FT* in apical buds^21^ during flower induction.

At microscopic scale, the first visible structural changes of the bud meristem appear during the second phase, the flower initiation. A pronounced doming and broadening of the bud apex is the first sign of floral commitment and marks the developmental onset of floral structures^1,22^. However, bud initiation within a given tree is synchronized to a certain extent by internal regulatory mechanisms and reported to persist for three to up to seven weeks^10,23^.

The subsequent flower bud differentiation, the third stage of the flower formation process, refers to the development of inflorescence primordia and floral organs and is temporarily interrupted with the onset of bud dormancy^14^. The formation of pollen sacs and ovules completes the bud differentiation in the following spring shortly before bud burst^24^.

Studying flower bud induction in apple has two main constraints. First, only time-dependent analyses of the bud cell structure provide a precise starting point of doming of the bud meristem, thus the transition from vegetative to floral meristems. Samples collected prior to this time point are then useful for studying the stage of flower induction. Second, composite samples of induced and non-induced buds are typically collected, yet this within-tree inhomogeneity introduces an experimental error for studying the underlying mechanisms of flower bud induction. However, by choosing extreme crop loads of heavy and no fruiting, there is a greater probability that all buds either remain vegetative or commit to flower formation. Consequently, in the current study, apple buds were collected from a biennial and non-biennial bearing apple cultivar with heavy and no crop, respectively, over two growing seasons and microscopically evaluated for determining the onset and duration of flower bud initiation in relation to heat accumulation. The results will define the starting point for RNAseq, proteome and metabolome analyses of the sampled apple buds prior to the identified flower initiation period. Moreover, we evaluated whether the reduction in return bloom following a high cropping year is the result of a delayed flower bud initiation and thus an incomplete flower bud development or fewer initiated buds in the preceding season.

## Materials and Methods

### Location and environmental conditions

Field experiments were conducted during the growing seasons in 2015 and 2016 at the Centre of Competence for Fruit Cultivation near Ravensburg, Germany (47°46′2.89″N 9°33′21.21″E, altitude 490 m). Climatic conditions in both years were typical for this fruit growing region. To evaluate the effect of temperature on flower bud initiation, the accumulated heat sum was calculated using the concept of Growing Degree Hours^25,26^ (GDH), using the following two equations:

1 $${GDH}={F}\frac{{{T}}_{{u}}-{{T}}_{{\boldsymbol{b}}}}{2}(1+\,\cos ({\pi }+{\pi }\,\ast \,\frac{{{T}}_{{h}}-{{T}}_{{b}}}{{{T}}_{{u}}-{{T}}_{{b}}}))$$

2 $${GDH}={F}({{T}}_{{u}}-{{T}}_{{b}})(1+\,\cos (\frac{{\pi }}{2}+\frac{{\pi }}{2}\,\ast \,\frac{{{T}}_{{h}}-{{T}}_{u}}{{{T}}_{c}-{{T}}_{u}}))$$

where T_h_ is the mean hourly temperature, T_b_ is the base temperature, T_u_ is the optimum temperature, T_c_ is the critical temperature and F is a plant stress factor that was set in both equations to 1. T_b_, T_u_ and T_c_ were set to 4 °C, 25 °C and 36 °C as suggested for fruit trees^25^. Equation 1 was used for mean hourly temperatures between T_b_ and T_u_, Eq. 2 was used for mean hourly temperatures between T_u_ and T_c_.

**Figure 1:**
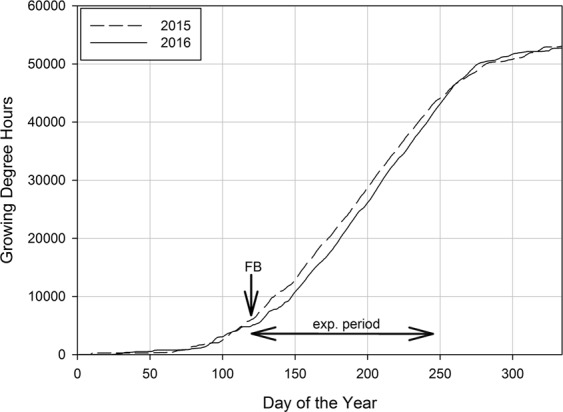
Growing degree hours (GDH) with a base temperature of 4 °C, accumulated from the beginning of each year. The experimental period started at full bloom (FB) and continued for 18 weeks.

Figure 1 shows the accumulated GDH with a base temperature of 4 °C calculated from the beginning of the year. From full bloom until the end of the experimental period at 18 weeks after full bloom, 2015 was with 37192 GDH warmer than 2016 with 36309 GDH.

### Experimental design

In this study, two apple cultivars with a different degree of biennial bearing behaviour were used. ‘Fuji’, clone ‘Raku-Raku’, is known to be a strongly biennial bearing cultivar, whereas ‘Gala’, clone ‘Galaxy’, is considered to bear more regularly. Both cultivars were grafted on M9 rootstock, planted in 2009 and trained as tall spindles of 4.5 m height. Standard orchard management practices, including winter pruning and fertilizer and pesticide applications, followed best practice guidelines. Date of full bloom, defined as the time point when at least 80% of all flowers are fully open, was 30 April 2015 and 27 April 2016 for both cultivars. This was determined by counting the number of open and closed flowers on selected branches of each cultivar. The experiment was carried out in two rows of ‘Fuji’ adjacent to two rows of ‘Gala’ with a tree spacing between rows of 3 m and within rows of 1 m. Trees were adjusted to commercial crop loads by mechanical and chemical thinning practices in previous years. At full bloom, 130 trees per cultivar were selected based on their flowering status within the two rows: 65 trees with a cultivar-specific low bloom density and 65 trees with a high bloom density, respectively. In 2015, the trees with low bloom density received the ‘off’ treatment by manually removing all flowers at full bloom, whereas trees with high bloom density received the ‘on’ treatment and were not flower-thinned. In 2016, the experimental trees were assigned to the opposite cropping treatment of the previous season to maintain the biennial bearing pattern, i.e. trees that were ‘off’ had all remaining flowers removed at full bloom and trees that were ‘on’ were not flower-thinned. Bloom density was expressed as number of flower clusters per trunk cross-sectional area (TCSA), calculated from the trunk circumference measured just prior to full bloom at 20 cm above the graft union^13^.

### Bud sampling

In 2015, bud sampling started in the fourth week after full bloom and continued for 15 weeks. Histological analysis of these buds indicated that bud initiation did not start before the eleventh sample was taken at 99 dafb. We therefore decided to shift the sampling period towards the seventh week after full bloom in 2016 and to shorten the sampling period to eight continuous weeks that should include both the stage of flower induction and the onset of flower initiation. An additional sample was taken 18 weeks after full bloom in 2016 to assess the bud initiation status near the end of the growing season.

At each sampling date, a new set of three trees per cultivar and treatment was randomly chosen and buds from 2-year-old spurs (≤5 cm) were collected from the periphery of the tree canopy. Sample size was one bud from each of three trees per cultivar and treatment at each sampling date whereas two buds from each of three trees per cultivar and treatment were taken for the last sample in 2016. In total 300 buds were collected for microscopy analysis. The tissue was immediately fixed in FAA^22^ (3.7% formaldehyde, 5% acetic acid, 50% ethanol) and stored at 4 °C until analysis.

### Bud microscopy

Buds were first dehydrated in a graded ethanol and xylol series and then infiltrated with paraffin at 58 °C. Each bud was first trimmed to one side before 100 median longitudinal sections of 5 µm thickness were prepared and stained with Wacker’s trichromatic W-3A botanical stain, containing acridine red, acriflavine and astra blue.

**Figure 2:**
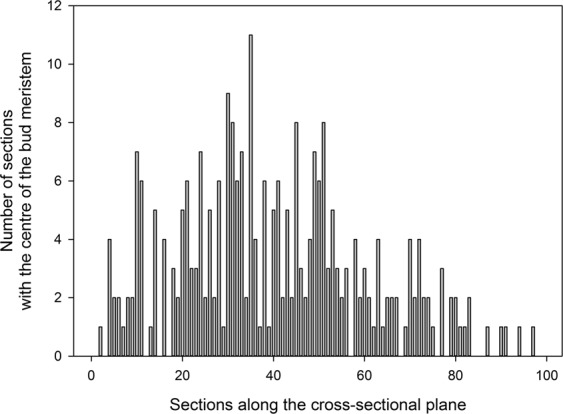
Number of sections that represented the centre of the bud meristem for each section along the cross-sectional plane (n = 268 buds).

**Figure 3:**
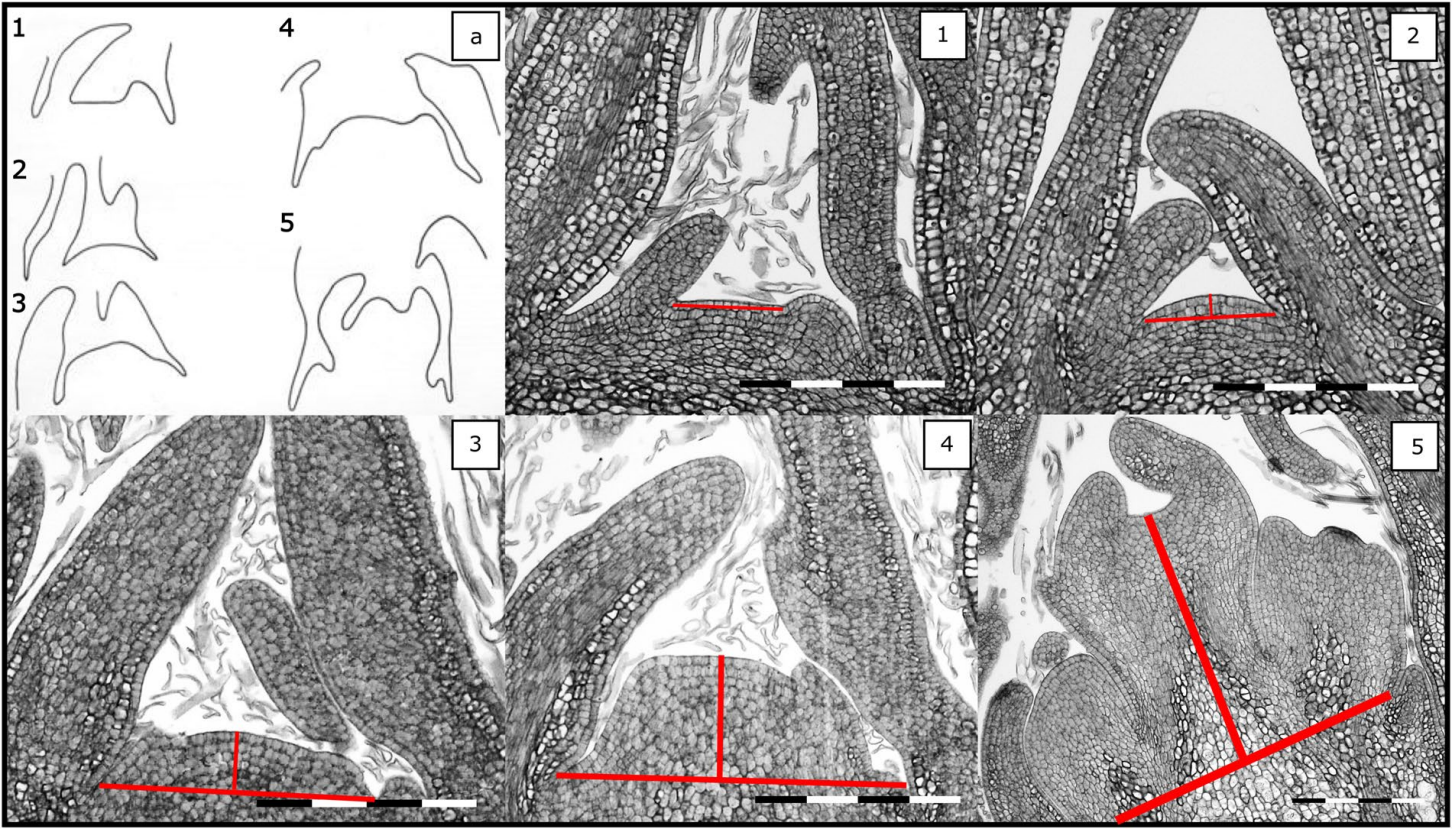
Five apple bud developmental stages. (a) Schematic representation of the five stages of floral bud development as previously shown in literature^27^; (1) narrow and flat vegetative apical meristem; (2) broad and swollen vegetative meristem; (3) doming of the apex as the first morphological sign of floral initiation; (4) formation of the inflorescence primordia; (5) differentiation of the inflorescence. Stages 1 and 2 are regarded as non-initiated, whereas stages 3 to 5 are considered as initiated. Full bar = 200 µm. Red lines indicate diameter and height of the meristem at each stage.

Consequently, 30,000 sections were prepared for determining ‘doming’ by selecting the centre of the meristem based on the parallel alignment of cells in the outer three tunica cell layers of the meristematic tissue. As shown in Fig. 2, the centre of the meristem was found in nearly all 100 sections, indicating that this detailed microscopy analysis is needed for detecting initiated buds. Bud developmental stages were determined following the classification^27^ shown in Fig. 3.

Developmental stages one and two were considered ‘non-initiated’ since it is not possible to determine microscopically whether the floral pathway has been triggered. In contrast, stages three to five were considered ‘initiated’ since the morphological characteristics of the meristems clearly indicate that floral development has started. The following binary dataset was used to model the probability of bud initiation in relation to heat accumulation. Furthermore, diameter and height of the bud apex were measured as shown in Fig. 3, using the image processing software ImageJ 1.50 g.

### Modelling the probability of bud initiation

Since the cultivars were not randomly allocated within the orchard block, trees per cultivar were considered as subsamples rather than true replicates. Consequently, no models were fitted with the factor cultivar since this main effect would have been confounded with position effects within the orchard. Accordingly, separate models for each cultivar were used to estimate the probability of buds being ‘initiated’ within a given sample in relation to heat accumulation since full bloom, using a logistic regression where the logarithm of the odds follows a linear model^28^.

The model for the cultivar ‘Gala’ (1) was only fitted to the 2015 data since bud initiation occurred after the last sampling date at 99 dafb in 2016:

1 $$\log (\frac{{{\pi }}_{{ij}}}{1-{{\pi }}_{{ij}}})={\mu }+{{\alpha }}_{{j}}+{{\beta }}_{1}{{x}}_{{i}}+\,{{\beta }}_{2{j}}{{x}}_{{i}}$$

where ${\pi }_{ij}$ denotes the probability of a bud being initiated at the *i*-th gdh of the *j*-th treatment; *µ* is the overall intercept; α_*j*_ represents the treatment-specific deviation from the common intercept for the *j*-th treatment; *β*_1_ is the common slope of the regression on growing degree hours accumulated from full bloom (gdh, *x*_*i*_); *β*_2*j*_ is the treatment-specific deviation from the common slope for the *j*-th treatment.

The model for the cultivar ‘Fuji’ (2) was fitted to the ‘off’ treatment since only one initiated bud was found in ‘on’ trees; thus no information was available to describe the bud initiation period in ‘on’ trees:

2 $$\log (\frac{{{\pi }}_{{im}}}{1-{{\pi }}_{{im}}})={\mu }+{{\gamma }}_{{m}}+{{\beta }}_{3}{{x}}_{{i}}+\,{{\beta }}_{4{m}}{{x}}_{{i}}$$

where ${\gamma }_{m}$ is the deviation from the common intercept $\mu$ for year *m*; ${\beta }_{3}$ is the common slope of the regression on growing degree hours; ${\beta }_{4m}$ is the year-specific deviation from the common slope for the m-th year.

Model terms were tested for significance by partial Wald-type χ²-tests. Throughout the entire statistical analysis, a significance level of α = 5% was used and non-significant interaction terms that indicated a common slope were removed from the model, following the common convention for keeping a model as simple as possible and to ease interpretation^28^.

For better visualization of the results, the model equations were back transformed from the logit-scale to the scale of response probabilities ${\pi }_{ij}$ (3), as shown exemplarily for ‘Gala’:

3 $${\pi }_{ij}(x)=\,\frac{{\exp }(\mu +{\alpha }_{j}+{\beta }_{1}{x}_{i}+\,{\beta }_{2j}{x}_{i})}{1+{\exp }(\mu +{\alpha }_{j}+{\beta }_{1}{x}_{i}+\,{\beta }_{2j}{x}_{i})}$$

To obtain the rate of initiation as a coefficient that describes the change of the probability per unit of heat accumulation, the response probability function was differentiated with respect to ${x}_{i}$ (4), as shown exemplarily for ‘Gala’:

4 $$\frac{d{\pi }_{ij}}{d{x}_{i}}=(\beta \,\ast \,(\frac{{\exp }(\mu +{\alpha }_{j}+{\beta }_{1}{x}_{i}+\,{\beta }_{2}{x}_{i})}{1+{\exp }(\mu +{\alpha }_{j}+{\beta }_{1}{x}_{i}+\,{\beta }_{2}{x}_{i})}))\,\ast \,(1-(\frac{{\exp }(\mu +{\alpha }_{j}+{\beta }_{1}{x}_{i}+\,{\beta }_{2}{x}_{i})}{1+{\exp }(\mu +{\alpha }_{j}+{\beta }_{1}{x}_{i}+\,{\beta }_{2}{x}_{i})}))$$

Equations (3) and (4) were also considered for ‘Fuji’ according to the model described in Eq. (2).

Logistic regression modelling was done using the LOGISTIC procedure in the software package SAS 9.4 (SAS Institute, Cary, USA).

## Results

**Figure 4:**
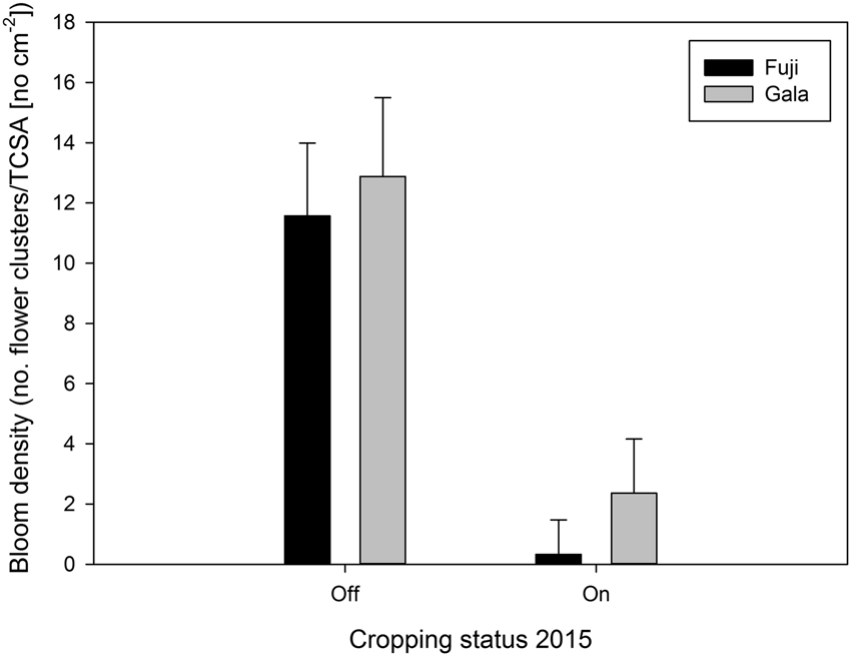
Bloom density expressed as mean (n = 65) number of flower clusters per trunk cross sectional area (cm²) in 2016 in response to the cropping status in 2015 for the cultivars ‘Fuji’ and ‘Gala’, respectively. Error bars indicate standard error.

Return bloom in 2016 was considerably greater on trees that were ‘off’ compared to ‘on’ in 2015 (Fig. 4). ‘Fuji’ ‘off’ trees had 35-fold more flower clusters per trunk cross sectional area than ‘Fuji’ ‘on’ trees, whereas ‘Gala’ ‘off’ trees carried only 5 times more flower clusters than ‘Gala’ ‘on’ trees. The return bloom in 2017 was rather similar to that found in 2016 (data not shown).

**Figure 5:**
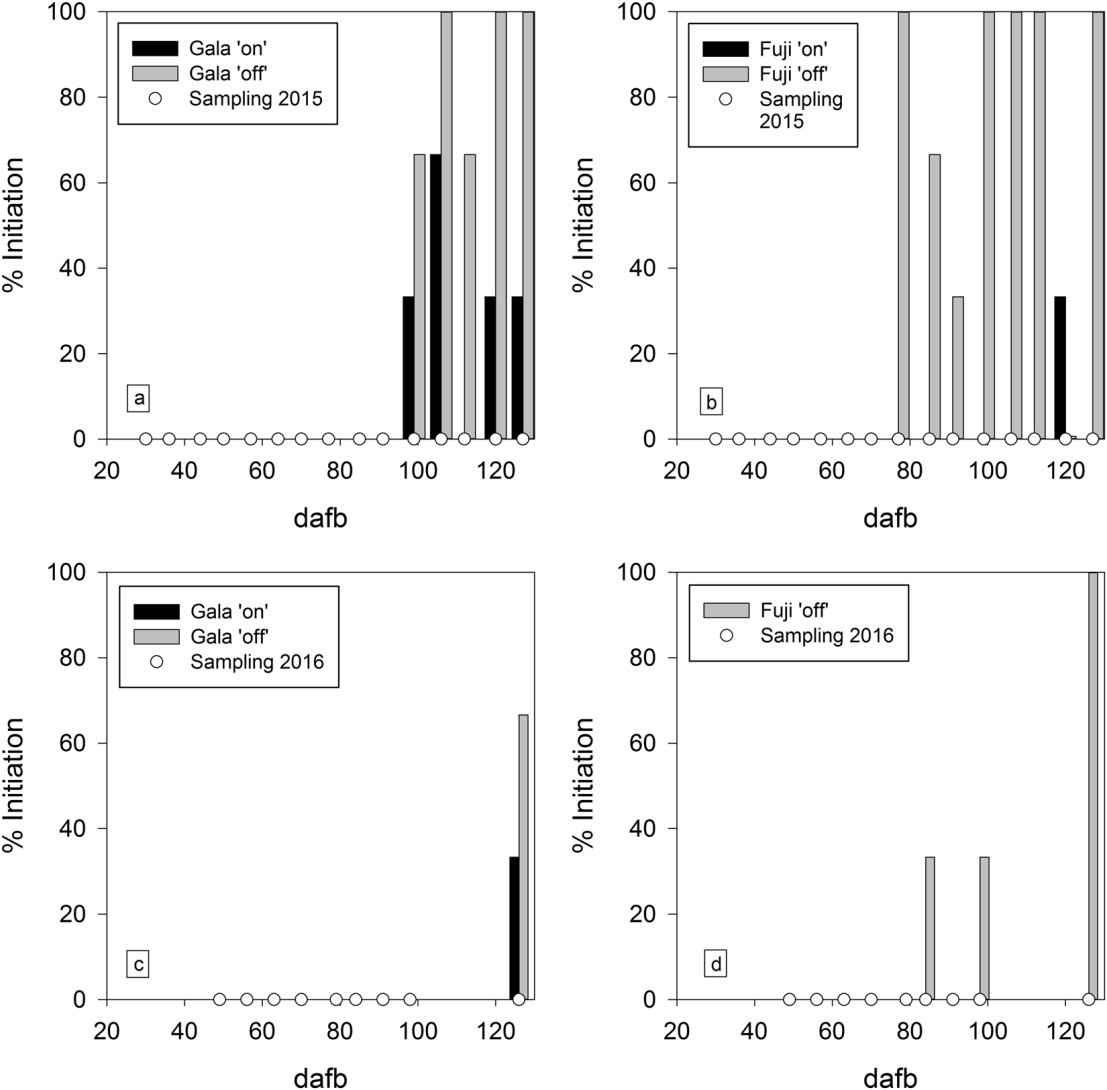
Mean percent bud initiation at days after full bloom, calculated separately at each sampling date, in ‘on’ and ‘off’ treatment, respectively, for the cultivars ‘Gala’ (**a** and **c**) and ‘Fuji’ (**b** and **d**) during two consecutive growing seasons. The ‘Fuji’ ‘on’ treatment is not shown since there was no initiated bud found in 2016.

In the 2015 growing season, the first initiated ‘Gala’ buds were found 99 dafb, irrespective of treatment, and bud initiation was observed until the last sampling date at 127 dafb (Fig. 5a). However, at each sampling time ‘off’ trees exhibited a much greater percentage of bud initiation than ‘on’ trees, resulting in average initiation percentages, calculated starting from the first sampling point where initiated buds were found until the last sampling date, of 87% and 33% for ‘off’ and ‘on’ trees, respectively. Bud initiation in ‘Fuji’ started in ‘off’ trees at 77 dafb, 22 days earlier than in ‘Gala’, but at 120 dafb in ‘on’ trees, yielding a mean percentage of initiation of 83% for ‘off’ trees and 17% for ‘on’ trees (Fig. 5b).

The first initiated ‘Gala’ buds in 2016 were noted at 126 dafb, again irrespective of treatment (Fig. 5c). Since no buds were collected between 98 and 126 dafb, bud initiation may have started earlier and nearer to the onset of bud initiation in 2015. Nevertheless, similar to 2015 results, ‘Gala’ ‘off’ trees showed a 2-fold higher percentage of initiation (66%) than ‘on’ trees (33%). In 2016, buds from ‘Fuji’ ‘off’ trees were first initiated at 84 dafb, which was close to the result in 2015; however, bud initiation was quite variable in subsequent samples. In contrast, buds from ‘Fuji’ ‘on’ trees were not initiated until the last sampling date (Fig. 5d).

The equations used to plot the probabilities of apple bud initiation as model outputs are given in Table 1.

**Table 1:** Equations for the probability of bud initiation and the initiation rate for the different cultivars, treatments and years.

	Probability of Initiation	Initiation Rate
Gala 2015 ‘off’	$y=\,\frac{\exp (-10.86+0.0004\,\ast \,x)}{1+\exp (-10.86+0.0004\,\ast \,x)}$	$y=(0.1162\,\ast \,(\frac{exp(-10.86+0.0004\,\ast \,x)}{1+exp(-10.86+0.0004\,\ast \,x)}))\,\ast \,(1-(\frac{exp(-10.86+0.0004\,\ast \,x)}{1+exp(-10.86+0.0004\,\ast \,x)}))$
Gala 2015 ‘on’	$y=\,\frac{\exp (-13.32+0.0004\,\ast \,x)}{1+\exp (-13.32+0.0004\,\ast \,x)}$	$y=(0.1162\,\ast \,(\frac{exp(-13.32+0.0004\,\ast \,x)}{1+exp(-13.32+0.0004\,\ast \,x)}))\,\ast \,(1-(\frac{exp(-13.32+0.0004\,\ast \,x)}{1+exp(-13.32+0.0004\,\ast \,x)}))$
Fuji ‘off’ 2015	$y=\,\frac{\exp (-8.00+0.0003\,\ast \,x)}{1+\exp (-8.00+0.0003\,\ast \,x)}$	$y=(0.1104\,\ast \,(\frac{exp(-8.00+0.0003\,\ast \,x)}{1+exp(-8.00+0.0003\,\ast \,x)}))\,\ast \,(1-(\frac{exp(-8.00+0.0003\,\ast \,x)}{1+exp(-8.00+0.0003\,\ast \,x)}))$
Fuji ‘off’ 2016	$y=\,\frac{\exp (-9.57+0.0003\,\ast \,x)}{1+\exp (-9.57+0.0003\,\ast \,x)}$	$y=(0.1104\,\ast \,(\frac{exp(-9.57+0.0003\,\ast \,x)}{1+exp(-9.57+0.0003\,\ast \,x)}))\,\ast \,(1-(\frac{exp(-9.57+0.0003\,\ast \,x)}{1+exp(-9.57+0.0003\,\ast \,x)}))$

Due to the sampling gap in 2016, equations for ‘Gala’ were only established for the 2015 dataset. The ‘Fuji’ ‘on’ treatment was not included since there was no (2015) and one initiated bud (2016) noted. *x* = heat accumulation from full bloom (growing degree hours). The interaction terms ‘gdh’*‘treatment’ for ‘Gala’ (p = 0.09) and ‘gdh’*‘year’ for ‘Fuji’ (p = 0.51) were dropped from the model because their p-values were above 0.05.

**Figure 6:**
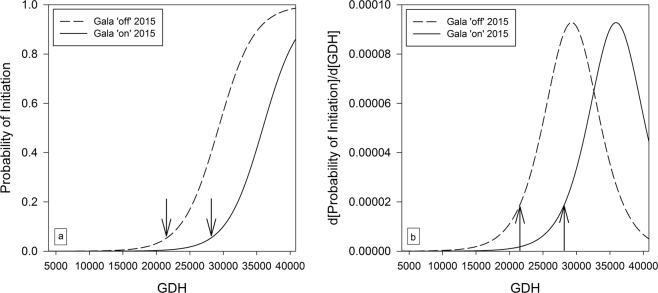
Modelled predictions of the probabilities of bud initiation (**a**) and the initiation rate (**b**) in ‘Gala’ ‘on’ and ‘off’ trees in 2015. Arrows indicate the onset of bud initiation defined as 20% of the maximum initiation rate: 21500 GDH (76 dafb) for ‘Gala’ ‘off’ trees in 2015; 28122 GDH (96 dafb) for ‘Gala’ ‘on’ trees in 2015.

The ‘Gala’ model could only be fitted to the data from 2015 due to sampling in 2016 not covering the bud initiation period. The main effects ‘gdh’ and ‘treatment’ were highly significant with p-values of 0.0002 and 0.0088, respectively. The interaction between ‘gdh’ and ‘treatment’ was removed from the model due to a non-significant effect in the Wald-χ²-test (p = 0.09). The onset of bud initiation, defined as 20% of the maximum initiation rate, occurred at 21500 GDH (76 dafb) for ‘off’ trees and at 28122 GDH (96 dafb) for ‘on’ trees. Both treatments had the same slope for the probability of bud initiation (Fig. 6a). Consequently, the maximum rate of bud initiation was also the same for both treatments (Fig. 6b) but it was reached 21 days earlier for ‘off’ (at 29282 GDH or 100 dafb) than for ‘on’ trees (at 35905 GDH or 121 dafb). The p-value for the Goodness-of-Fit (GOF) Test of Hosmer and Lemeshow^29^ was 0.9, indicating a high goodness of fit for the model.

**Figure 7:**
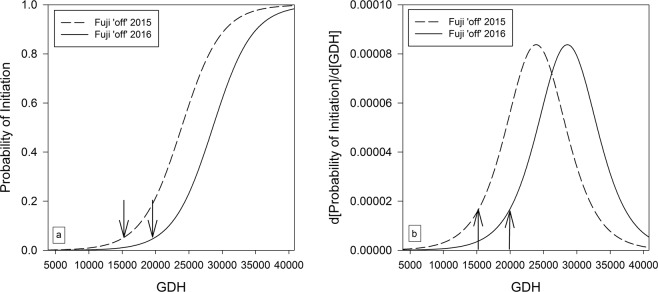
Modelled predictions of the probabilities of bud initiation (**a**) and the initiation rate (**b**) in ‘Fuji’ ‘off’ trees in 2015 and 2016. Arrows indicate the onset of bud initiation defined as 20% of the maximum initiation rate: 15274 GDH (57 dafb) for ‘Fuji’ ‘off’ trees in 2015; 19955 GDH (72 dafb) for ‘Fuji’ ‘off’ trees in 2016.

Since only one initiated bud was found for ‘Fuji’ ‘on’ trees, modelling this treatment was not possible. Thus, Fig. 7 shows the predictions of the probabilities of bud initiation and the initiation rate in ‘Fuji’ ‘off’ trees for both consecutive growing seasons. The main effect ‘gdh’ was highly significant with a p-value of 0.001, whereas the effect ‘year’ was not significant with a p-value of 0.0718. The interaction between ‘gdh’ and ‘year’ was dropped from the model since a non-significant effect was found in the Wald-χ²-test (p = 0.51). The onset of bud initiation for ‘Fuji’ ‘off’ trees was 15274 GDH (57 dafb) in 2015 and 19955 GDH (72 dafb) in 2016. Both years had the same slope for the probability of bud initiation (Fig. 7a), resulting in an identical maximum rate of bud initiation that was reached 18 days earlier in 2015 (at 23893 GDH or 84 dafb) than in 2016 (at 28574 GDH or 102 dafb) (Fig. 7b). The p-value for the GOF Test of Hosmer and Lemeshow^29^ was again not significant with 0.09, indicating a reasonable goodness of fit for the model.

The length of the active initiation period, defined as the period with a rate of initiation of at least 20% of the maximum rate, was 49 days in ‘Gala’ ‘off’ trees in 2015, 63 days in ‘Gala’ ‘on’ trees in 2015, 53 days in ‘Fuji’ ‘off’ trees in 2015 and 2016, respectively. However, the maximum initiation rate was 5% lower in ‘Fuji’ ‘off’ trees compared to ‘Gala’ ‘off’ trees. A continued decline of the bud initiation rate to 20% was not observed in ‘Gala’ ‘on’ trees in 2015, because the sampling period ended shortly after the maximum rate was obtained.

**Figure 8:**
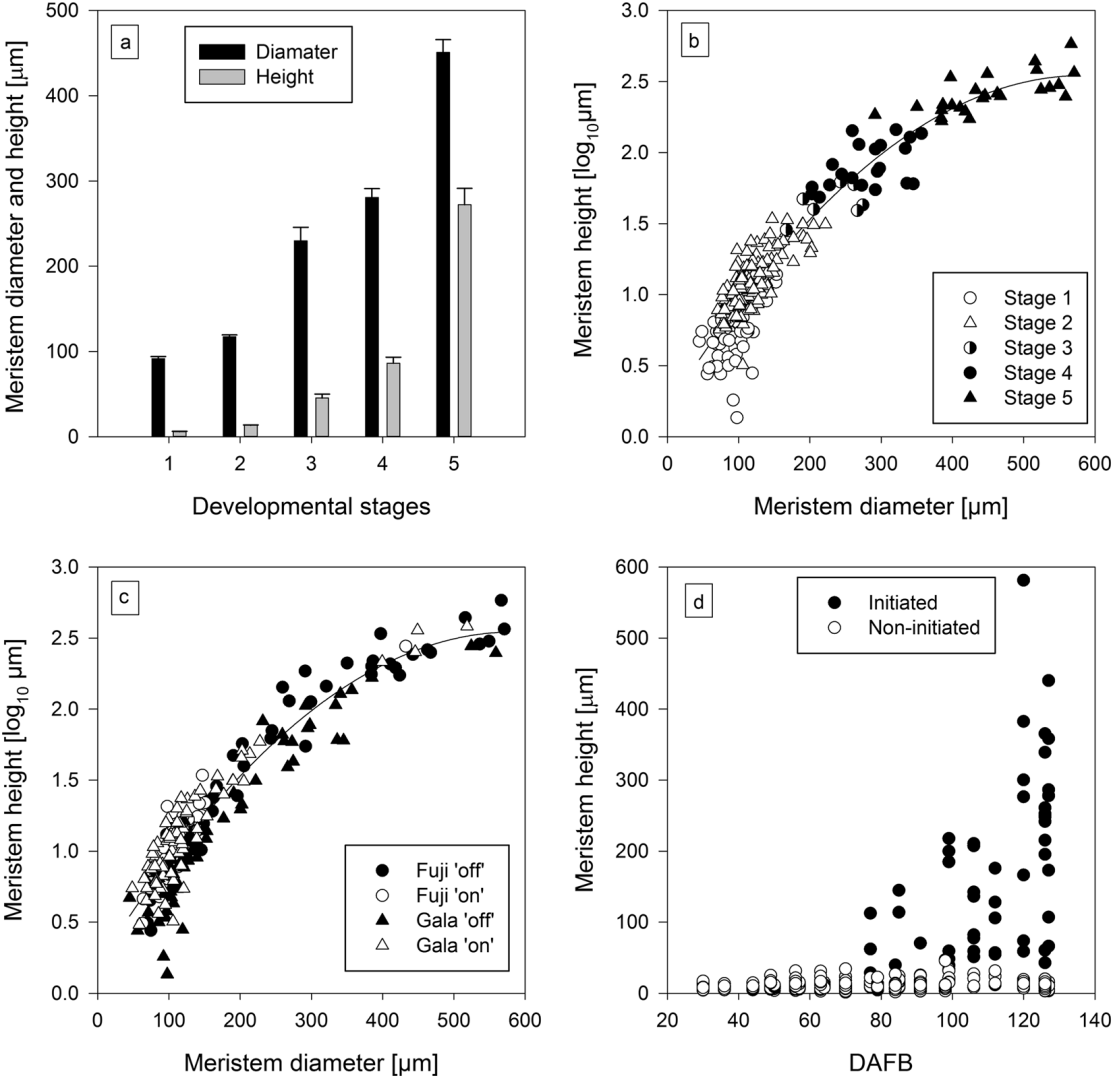
Mean height and diameter of bud meristems at each developmental stage across both cultivars and treatments (vertical bars are standard error) (**a**), relationship between meristem diameter and height at each developmental stage (**b**) as well as for each cultivar and treatment combination (**c**), and the time-dependent pattern of meristem height of initiated and non-initiated buds across both cultivars and treatments (**d**). All figures are based on data from 2015 and 2016. Equation for the regression line in (**b,c**): y = −6.8723E-006x² + 0.008x + 0.1975 with R² = 0.899.

The size of bud meristems was distinctly different between initiated and non-initiated buds (Fig. 8). Stages 1 and 2, considered vegetative, did not vary considerably in height and diameter of the meristem (Fig. 8a), whereas the later stages, characterized by floral commitment, had larger meristems. Initiated buds had meristems with a diameter of at least 200 µm and a height of at least 32 µm (Fig. 8a,b). A regression analysis of all bud meristems in both experimental years suggested that meristem diameter is strongly correlated to meristem height (R² = 0.89), irrespective of the dataset sorted by meristem stage (Fig. 8b) or cultivar and crop load combination (Fig. 8c). The meristem height of initiated buds increased continuously from 77 dafb, the beginning of doming, until the last sample at 127 dafb, whereas it remained at constant level for non-initiated buds throughout that period (Fig. 8d).

## Discussion

### Meristem characteristics

Measuring the meristem size is a useful tool to describe the distinct morphological characteristics of initiated and non-initiated buds, which was also reported in a study assessing the development of buds in ‘Starkspur Supreme Delicious’ apple^30^. The beginning of doming at 77 dafb is in agreement with findings from other studies^22,31^, which observed pronounced doming in the majority of apple bud samples between 72 and 127 dafb. A bimodal distribution of vegetative meristem diameters as reported by others^22^ was not observed in this study.

The non-observed initiation of buds in ‘Fuji’ ‘off’ trees at 91 dafb (Fig. 5d) indicates some degree of variability in floral bud development among sample dates and this biologically expected heterogeneity was also found in other studies^32,33^.

### Crop load

The model for the ‘Gala’ 2015 dataset shows, that the onset of floral bud initiation but not the rate of initiation was significantly affected by the ‘on’ and ‘off’ crop load treatments, respectively. Thus, under the specific climatic conditions in southwest Germany, trees bearing no fruit started bud initiation on 2-year-old spurs at 76 dafb, whereas this developmental process was delayed in heavy cropping trees until 96 dafb. This result is not in agreement with findings of other authors who reported that the period of flower bud induction begins sooner in ‘on’ than in ‘off’ trees^34^ or showed that the presence of fruit inhibited flower bud formation earlier on trees with heavy bloom than on those with light bloom^17^. However, the results show that environmental factors and tree cropping status may explain the largely ambiguous and inconsistent published data for the onset of floral bud initiation in apple^23^.

Despite an 80% probability of bud initiation in ‘Gala’ ‘on’ trees at the end of the experimental period (Fig. 6a), return bloom in the following year was relatively small with just over 2 flower clusters cm^−2^ TCSA. We postulate that heavy cropping delays the onset of flower bud initiation to such extent that floral bud differentiation is poorly advanced prior to endodormancy, leading to little return bloom.

### Cultivar

Although the experimental design did not allow for a statistical evaluation of cultivar differences, the data substantiate the commonly accepted notion that the genetic make-up of a cultivar profoundly affects the onset of flower bud initiation^31^. For example, the onset of bud initiation on ‘off’ trees was 57 dafb in ‘Fuji’ and 76 dafb in ‘Gala’ in 2015. A difference in floral initiation of approximately 19 days between ‘Fuji’ and ‘Gala’ was also found under the climatic conditions in New Zealand^31^; however, doming occurred much later in ‘Fuji’ (86 dafb) and ‘Gala’ (112 dafb). In another study in New Zealand^23^, the onset of floral bud initiation for ‘Royal Gala’ was between 72 to 99 dafb, which is similar to that of heavy cropping ‘Gala’ trees at 96 dafb in southwest Germany in our study.

The percentages of bud initiation (Fig. 5) were studied for trees with an extremely high and no crop load, respectively. The treatment dependent differences found in this study may be reduced by thinning practices usually applied in commercial apple orchards. Indeed, appropriate thinning techniques can effectively regulate the percentage of bud initiation even in strongly biennial bearing cultivars^35^. However, in a different study (unpublished data) with three apple cultivars thinned to a medium crop load (approx. 4 fruit cm^−2^ TCSA), the percentage of initiated buds at 126 dafb was 68% for ‘Fuji’, 71% for ‘Gala’ and 98% for ‘Braeburn’. Although no significant differences were found, a clear cultivar-specific trend in floral bud initiation was evident. Moreover, the tree to tree variability in the percentage of initiated buds was much larger in ‘Fuji’ and ‘Gala’ than in ‘Braeburn’. These results are in agreement with other findings^36^, where a heterogenous biennial bearing behaviour among trees of the same cultivar within the same orchard was found.

### Heat accumulation

The onset of initiation for ‘Fuji’ ‘off’ trees occurred 5 days after summer solstice in 2015 (June 26) but 20 days after summer solstice in 2016 (July 11). The observation that flower bud initiation did not occur on the same day in both years supports the notion by other authors that flower bud initiation cannot be entirely and fully deterministically driven by daylength^10,37,38^.

The difference in the onset of bud initiation for ‘Fuji’ ‘off’ trees between 2015 (15274 GDH or 57 dafb) and 2016 (19955 GDH or 72 dafb) was 23% for GDH or 21% for dafb (Fig. 7). GDH accumulated from full bloom explained better the existing annual variability in the onset of bud initiation than GDH from the transition from short- to long-day or GDH with a base temperature of 10 °C as used in another study^23^. Moreover, the probabilities of bud initiation in both years nearly merge at approximately 40000 GDH, indicating some degree of recovery following the delayed onset of bud initiation in 2016. The results suggest that heat accumulation has a modulating effect on the pattern (onset, progression, termination) of bud initiation. There is some experimental evidence^39^, that flower bud initiation is advanced under higher temperatures; however, this was not supported by our results.

The difference in the onset of bud initiation in ‘Gala’ ‘off’ (21500 GDH or 76 dafb) and ‘Gala’ ‘on’ trees (28122 GDH or 96 dafb) in 2015 was clearly due to treatment effects and was 23.5% for GDH (Fig. 6) and 21% for dafb. If an earlier onset of flower bud initiation in heavy cropping trees would solely require a higher temperature demand, the onset in ‘Gala’ ‘on’ trees would occur at a similar time as ‘off’ trees when accumulating 23.6% more GDH 20 days earlier. However, it is more likely that a minimum temperature accumulation is required for triggering the onset of bud initiation, but a crop load mediated factor is delaying this process. This factor might be a decreased carbohydrate supply of the bud meristems in heavy cropping trees as already suggested in literature^6^. Although the total carbohydrate requirement for flower bud formation may be small compared with that of other sinks (e.g. fruit), there is now growing evidence that the proportion of carbohydrates available for flower bud formation might be limited due to a low priority rank order of buds and/or exhausted carbohydrate reserve pools. Excessive cropping in the ‘on’ year may reduce plant carbohydrate reserves and this deficiency may exert a carbohydrate-induced inhibitory effect of source limitation on flower bud induction.

## Conclusion

**Figure 9:**
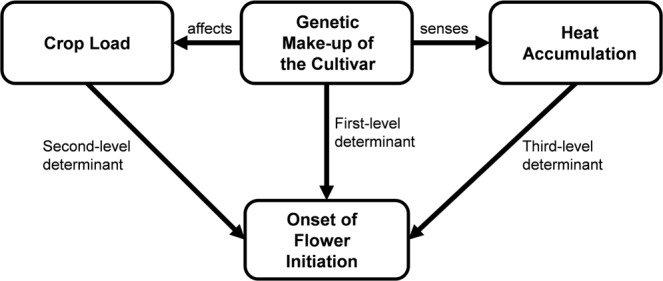
Proposed factors affecting the onset and duration of flower initiation in apple: the genetic make-up of the cultivar, crop load and heat accumulation.

The results from this study can be summarised in a proposed concept (Fig. 9), which identifies the main factors influencing the onset of flower bud initiation in apple. In general, the genetic make-up of a given cultivar is the first-level determinant for the onset of flower bud initiation as indicated by the large differences between ‘Fuji’ ‘off’ and ‘Gala’ ‘off’ trees in 2015, despite being exposed to the same heat accumulation and crop load conditions. High crop load delayed considerably the onset of bud initiation in ‘Gala’, a response that may occur irrespective of cultivar. However, this response could not be modelled for the strongly biennial bearing cultivar ‘Fuji’ due to the absence of initiated buds in ‘on’ trees. It remains unclear whether this crop load driven (second-level determinant) response in the onset of flower bud initiation is temperature-dependent. Nevertheless, the yearly differences in the onset of bud initiation are related to differences in heat accumulation (third-level determinant). To study the interactions between cultivar, crop load and temperature in relation to flower bud initiation requires specific experimental setups and can only be fully investigated in controlled-environment chambers.
